# Ruthenium-Anchored Carbon Sphere-Customized Sensor for the Selective Amperometric Detection of Melatonin

**DOI:** 10.3390/bios13100936

**Published:** 2023-10-18

**Authors:** Sivaguru Jayaraman, Thenmozhi Rajarathinam, Hyeon-Geun Jang, Dinakaran Thirumalai, Jaewon Lee, Hyun-Jong Paik, Seung-Cheol Chang

**Affiliations:** 1 Department of Cogno-Mechatronics Engineering, College of Nanoscience and Nanotechnology, Pusan National University, Busan 46241, Republic of Korea; sivaguru@pusan.ac.kr (S.J.); thenmozhi@pusan.ac.kr (T.R.); tim4545@pusan.ac.kr (H.-G.J.); 2 BIT Convergence-Based Innovative Drug Development Targeting Metainflammation, Pusan National University, Busan 46241, Republic of Korea; dinakaran@pusan.ac.kr (D.T.); neuron@pusan.ac.kr (J.L.); 3 Department of Pharmacy, College of Pharmacy, Pusan National University, Busan 46241, Republic of Korea; 4 Department of Polymer Science and Engineering, Pusan National University, Busan 46241, Republic of Korea; hpaik@pusan.ac.kr

**Keywords:** melatonin, amperometric sensor, ruthenium, carbon spheres, lower detection limit, pharmaceutical samples

## Abstract

Melatonin (MT), a pineal gland hormone, regulates the sleep/wake cycle and is a potential biomarker for neurodegenerative disorders, depression, hypertension, and several cancers, including prostate cancer and hepatocarcinoma. The amperometric detection of MT was achieved using a sensor customized with ruthenium-incorporated carbon spheres (Ru–CS), possessing C- and O-rich catalytically active Ru surfaces. The non-covalent interactions and ion–molecule adducts between Ru and CS favor the formation of heterojunctions at the sensor–analyte interface, thus accelerating the reactions towards MT. The Ru–CS/Screen-printed carbon electrode (SPCE) sensor demonstrated the outstanding electrocatalytic oxidation of MT owing to its high surface area and heterogeneous rate constants and afforded a lower detection limit (0.27 μM), high sensitivity (0.85 μA μM ^−1^ cm^−2^), and excellent selectivity for MT with the co-existence of crucial neurotransmitters, including norepinephrine, epinephrine, dopamine, and serotonin. High concentrations of active biomolecules, such as ascorbic acid and tyrosine, did not interfere with MT detection. The practical feasibility of the sensor for MT detection in pharmaceutical samples was demonstrated, comparable to the data provided on the product labels. The developed amperometric sensor is highly suitable for the quality control of medicines because of its low cost, simplicity, small sample size, speed of analysis, and potential for automation.

## 1. Introduction

Melatonin (MT), with the chemical name *N*-acetyl-5-methoxytryptamine, is a tryptophan-derived lipophilic hormone produced and secreted at night by the pineal gland. MT is a multifunctional pleiotropic neurohormone that protects against neurotoxicity and oxidative stress [1,2]. MT modulates the sleep/wake cycle (circadian rhythmicity), retinal and immune functions and is thus commonly used as a medication for insomnia and jetlag. MT has a characteristic circadian-synthesis profile with high levels at night and low levels throughout the day, in synchrony with the natural cycle of light and dark [3,4]. Some studies have reported that high oscillation of MT scores in humans are linked to various neurodegenerative disorders, such as Alzheimer’s disease, Parkinson’s [5,6], Huntington’s disease, depression [7], and hypertension [8]. MT is also a potential biomarker for the diagnosis of prostate cancer or hepatocarcinoma [9]. Moreover, MT is an antioxidant and free radical scavenger. Owing to the high clinical relevance of MT, the development of an accurate estimation technique is essential for diagnosing and understanding the biological relationship between MT and the aforementioned disorders [10]. Notably, an error-free/precise technique for investigating MT levels can ensure easy applicability in the quality control of medicines.

Over the past decades, MT analysis has gained increasing significance, where confirmatory techniques, such as radioimmunoassay [11], calorimetry [12], enzyme-linked immunoassay [13], high-resolution nuclear magnetic resonance spectroscopy [14], high-performance liquid chromatography [15,16], mass spectrometry [17,18], and electrochemical approaches [19], have been used to detect MT levels. However, owing to the low concentrations of MT (0.043 μM in the day and 0.86 μM in the night [20]) and co-existence of several endogenous metabolites in biological fluids, the accurate detection of MT at low concentrations with sufficient anti-interference ability remains a challenge. Among the techniques used for MT detection, electrochemical techniques are reported to be ecofriendly, require low manpower handling, are less susceptible to matrix effects, and enable highly sensitive and selective real-time detection with rapid responses [19].

Carbon nanomaterials, especially carbon nanospheres (CSs), are unique electrocatalysts for fabricating electrochemical sensors owing to their high conductivity, large specific surface area, well-controlled pore systems, concise synthetic routes, and easy functionalization. Carbon nanomaterials also provide active sites for electron transfer and enable efficient mass transport [21,22]. Especially, the C and O functionalities promote electron-transfer kinetics [23]. The encapsulation or confinement of metal catalysts in a porouscarbon matrix results in substantial improvements in the catalytic performance compared to that of the unencapsulated congeners. Thus, the self-aggregation of metal catalysts is prevented, and their stability is improved in the porous carbon matrix [24]. In the past decade, noble metal (Au, Ag, and Pt)-loaded CSs have received extensive research attention owing to its unique morphology and potential as an electrocatalyst [25,26,27,28] in sensor fabrication [26]. Noble metals promote the diffusion of target molecules towards the active metal sites; however, they suffer from the drawbacks of low metal dispersion and metal leaching. Ruthenium (Ru), a transition metal that is inert to most other chemicals, has been employed to overcome these drawbacks. Ru has emerged as a substitute for noble metals, such as rhodium, iridium, gold, silver, and platinum, because it is more cost-effective than noble metals [29]. Ru has been reported to exhibit a peroxidase-like property and superior chemical stability on exposure to aqua regia [30]. In addition, the C- and O-rich Ru surface is catalytically active [31], and interfacial contacts between Ru and C atoms further facilitate relatively strong interactions with the target, thus accelerating the reactions of the target at the electrode and preventing Ru aggregation, leaching, surface oxidation, and cross-coupling reactions [32]. As a polyvalent metal, Ru can easily adopt various oxidation states, leading to the formation of a multitude of complexes with unique properties [33]. Different configurations of Ru in various phases alter the electrocatalytic activity and electronic and geometric structures. Ru is highly intriguing for electrochemical sensing applications owing to its multifarious intrinsic properties, including its tunable structure, high electrical conductivity, high surface area, reduced resistance, and improved accessibility to electrolyte ions [34]. These excellent features inspired us to explore the catalytic activity of Ru nanoparticle (NP)-loaded CSs.

Herein, we report an efficient, rapid, and cost-effective electrochemical sensor employing Ru-loaded CSs for MT detection. The concept is based on the use of polydopamine (pDA) as a carbon source, which takes advantage of the inherent ability of pDA to interact with metal ions, forming ion–molecule adducts [35]. Initially, Ru–CSs are synthesized via the polymerization of dopamine (DA) to pDA, condensation, and subsequent high-temperature calcination of the formed Ru–pDA to Ru–CSs. Screen-printed carbon electrodes (SPCEs) are subsequently modified with Ru–CSs for detecting MT through chronoamperometry (CA). The fabricated Ru–CS/SPCE sensor displays a low limit of detection (LOD = 0.27 µM) due to the large active surface area between the Ru–CS and MT molecules. The present study has the following merits. (1) CSs are synthesized utilizing a simple carbon source, pDA. (2) the size and dispersion of the Ru NPs on CSs are finely tuned through condensation and high-temperature calcination processes, and (3) for the first time, Ru–CSs are employed as an electrocatalyst for the electrochemical detection of MT in pharmaceutical samples.

## 2. Materials and Methods

### 2.1. Materials

The following chemicals were procured from Sigma-Aldrich (Seoul, Republic of Korea): aqueous ammonia solution (1.0 mL, 25–28%), dopamine hydrochloride (DA), potassium ferricyanide and ferrocyanide [Fe(CN)_6_]^3−/4−^, potassium chloride (KCl), hydrochloric acid (HCl, 37%), concentrated sulfuric acid (H_2_SO_4_), ethanol (99.5% purity), toluene (99.5%), and ruthenium(III) chloride (RuCl_3_). MT, ascorbic acid (AA), tyrosine (Ty), norepinephrine (NE), epinephrine (EP), and serotonin (5–HT), were used for the interference studies. Phosphate buffers (0.1 M) were prepared by following a standard protocol [36]. All aqueous solutions were prepared using 18.2 MΩ water.

### 2.2. Synthesis of CSs and Ru–CSs

DA was polymerized to polydopamine (pDA) via a condensation process and used in the subsequent synthesis of the Ru–CS materials. The synthesis procedure was a modification of a previously reported method [37]. For CS synthesis, ammonia (1.0 mL, 25–28%) was mixed with ethanol (18 mL) and 18.2 MΩ.cm water (48 mL) under stirring for 30 min at 300 rpm; this mixture was labeled as Solution A. DA (250.0 mg) was dissolved in ethanol (2 mL) and 18.2 MΩ water (3 mL), with the solution being stirred at 300 rpm for 30 min, and labeled as Solution B. Solution B was then slowly injected into Solution A, under stirring for 12 h at 60 °C. CSs were obtained through the condensation process in a Teflon-lined steel autoclave at 210 °C for 12 h, which was then separated via filtration and washed thrice with 18.2 MΩ water and ethanol. The CS product was washed and re-dispersed in ethanol. Finally, the product was calcined at 600 °C for 6 h under an N_2_ atmosphere.

**Scheme 1:**
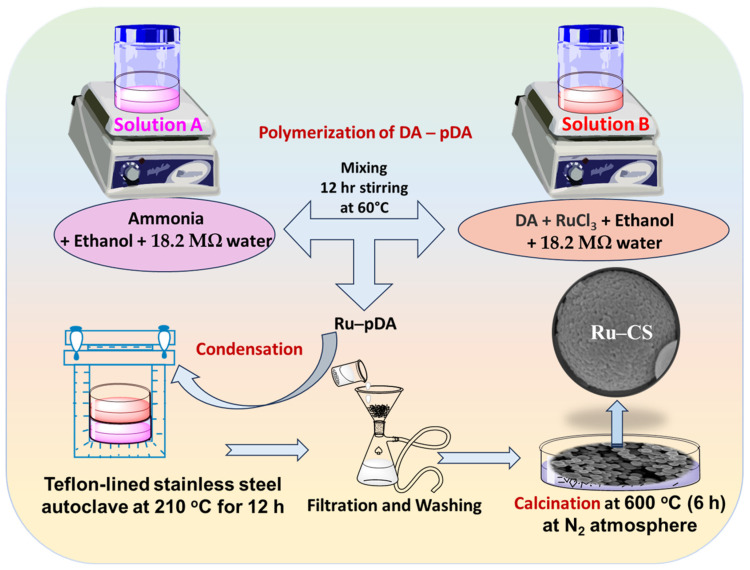
Synthesis of Ru–CSs through the polymerization of DA to pDA, condensation, and calcination processes under a N_2_ atmosphere.

For Ru–CS synthesis, an additional Solution A was prepared. Subsequently, DA (250.0 mg) and RuCl_3_ (2.8 mg, 0.2 mol% to DA) were dissolved in a mixture of ethanol (2 mL) and 18.2 MΩ water (3 mL); the resulting mixture was stirred at 300 rpm for 30 min and labeled as Solution B. Solution B was then mixed/injected into Solution A, under stirring for 12 h at 60 °C. Ru–CSs were obtained through the condensation of Ru–pDA in a Teflon-lined steel autoclave at 210 °C for 12 h. The resulting Ru–pDA was separated via filtration and washed thrice with 18.2 MΩ water and ethanol. The acquired product was washed and re-dispersed in ethanol. Finally, the product was calcined at 600 °C for 6 h under an N_2_ atmosphere. In this process, Ru ions were reduced to Ru NPs (Scheme 1). The Ru NPs confined within the CSs were expected to enhance the catalytic reaction of the sensor towards the target.

### 2.3. Fabrication of Ru–CS/SPCE

SPCEs were purchased from Metrohm DropSens (C11L, Oveido, Spain), and they were modified with 6 µL of Ru–CSs (1.0 mg mL^−1^) via a simple drop-casting technique and dried at the room temperature of 25 °C. The modified Ru–CS/SPCE was utilized as a working electrode. Integrated Ag/AgCl and platinum electrodes were used as reference and counter electrodes.

### 2.4. Instrumentation and Measurements

Detailed information on the instruments used and their measurement techniques are provided in Section S1 of the Supplementary Material.

### 2.5. Preparation of Pharmaceutical Samples

The applicability of the sensor was studied by analyzing pharmaceutical-grade melatonin samples. The pharmaceutical samples of melatonin, Cosmopharm melatonin (3 mg), and Nature Made Melatonin (5 mg), were procured from a local store and Nature Made Nutritional Products, West Hills, Los Angeles, CA, USA.

The pharmaceutical samples were ground to a fine powder using a mortar and pestle, homogenized, and dissolved in a phosphate buffer solution under ultrasonication for 30 min. After filtration, the final filtrate was again diluted with a phosphate buffer solution. Aliquots of this solution were withdrawn for CA measurements. Each pharmaceutical sample was prepared in several dilutions (1:10, 1:50, and 1:100) and analyzed in triplicate.

## 3. Results and Discussion

### 3.1. Morphology/Microstructure and Elemental Analysis

**Figure 1:**
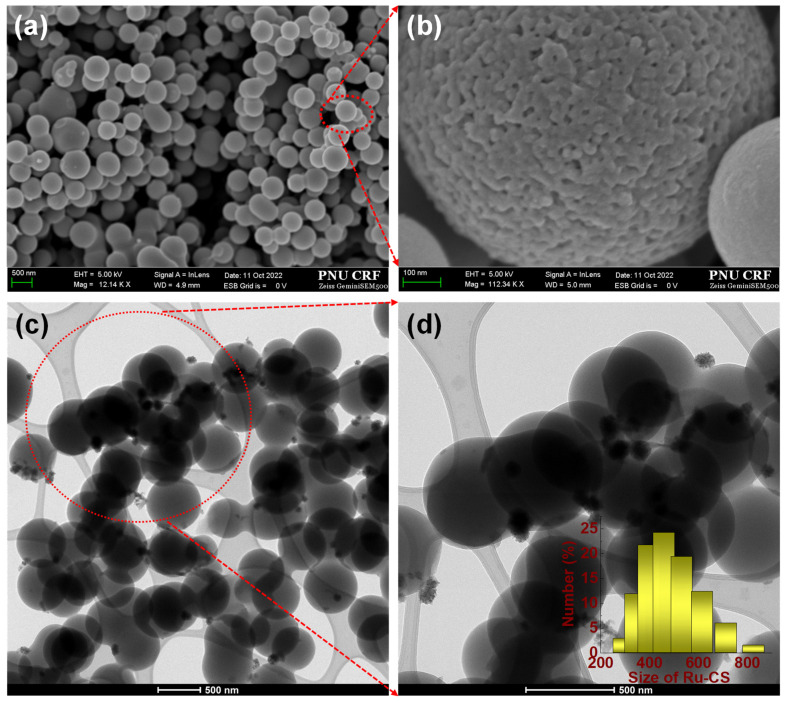
FE–SEM images of (**a**) Ru–CSs at a 500 nm scale; the red circle indicates the area in the magnified image in (**b**) at a 100 nm scale. FE–TEM images of (**c**) Ru–CSs at 500 nm scale; the red circle indicates the area in the magnified image in (**d**) at a 500 nm scale. The inset is the average particle size of Ru–CSs.

**Figure 2:**
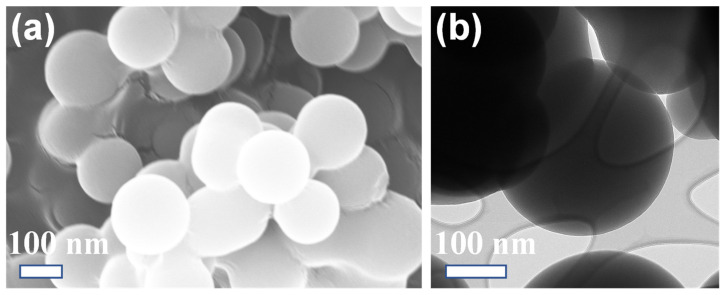
(**a**) FE–SEM and (**b**) TEM images of (**a**) CSs at a 100 nm scale.

**Figure 3:**
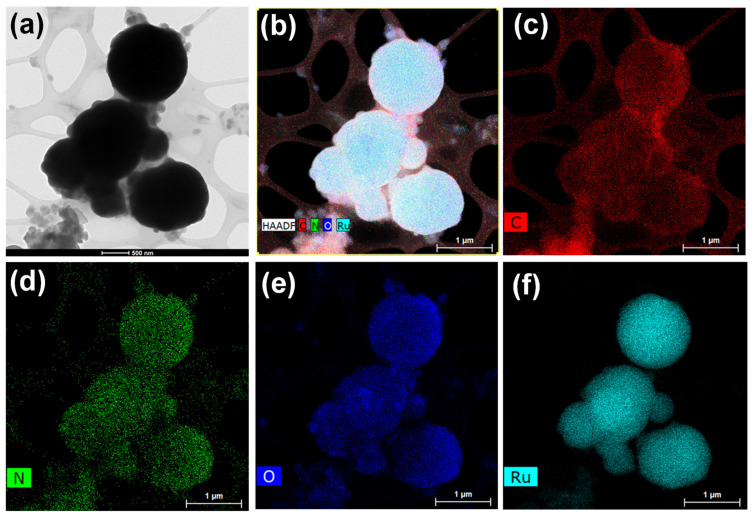
(**a**) TEM image of Ru–CSs at a 500 nm scale. (**b**) HAADF-STEM image of Ru–CSs showing the presence of C, N, O, and Ru at a 1 µm scale; (**c**–**f**) corresponding individual elemental maps of C, N, O and Ru.

The textural properties of the synthesized Ru–CSs and CSs were explored via field-emission scanning electron microscopy (FE–SEM) and transmission electron microscopy (TEM), as shown in Figure 1, Figure 2 and Figure 3. The Ru–CS (Figure 1a) particles had uniform, spherical shapes with a much smoother surface owing to impregnation of the Ru NPs. The enlarged image in Figure 1b shows a perfect Ru–CS sphere with a porous microstructure. The TEM images of Ru–CSs in Figure 1c,d confirm the presence of very small and uniform Ru NPs trapped on the spherical particles of CSs. The average particle size of Ru–CSs was around 500–600 nm (Figure 1d inset). The FE–SEM image of CSs also exhibited a uniform, spherical morphology, whereas the surface was slightly rough with minor agglomeration between the spheres, which can be ascribed to slight polymerization interruptions (Figure 2a), similar to a previous report [28]. The TEM image of CSs in Figure 2b also reveals a perfectly spherical morphology.

High-angle annular dark-field scanning transmission electron microscopy (HAADF–STEM) and energy-dispersive X-ray (EDX) mapping revealed that the C, N, O, and Ru atoms were uniformly distributed throughout the spheres (Figure 3).

The surface composition and chemical valence states of Ru in the synthesized Ru–CSs were examined through X-ray photoelectron spectroscopy (XPS) measurements. Clearly, the XPS survey spectra represented in Figure S1a reveal the presence of elements, such as C, N, O, and Ru elements, at the binding energies of 284.0 eV, 399.0 eV, 531.7 eV, and 460.7 eV, respectively. The deconvoluted Ru3p spectra (Figure S1b) speculated on the presence of two distinct Ru peaks, Ru 3p_1/2_ and Ru 3p_3/2_, at the binding energies of 461.7 eV and 486.2 eV, respectively. Both the Ru 3p_1/2_ and Ru 3p_3/2_ peaks evidenced the presence of the mixed valence states of Ru, namely the Ru^0^ and Ru^2+^ states. The sub-peaks centered at 461.5 eV and 483.7 eV are assigned to the electron-richer Ru^0^ states, while the sub-peaks centered at 463.8 eV and 486.1 eV are indexed to the oxidized Ru^2+^ species. The optimal Ru (Both Ru^0^ and Ru^2+^):C ratio was 1:39. The interfacial bonding interaction is probably dominated by d electrons from the Ru metal core donated to the π* orbitals of C≡N, rather than the donation of the N lone-pair electrons to Ru, similar to the previous report [38]. Thus, the formation of strong interfacial contacts between Ru and N, as well as Ru and C, verify the formation of ion–molecule adducts between Ru and CSs.

Further, the atomic % value of C, N, O, and Ru were found to be 70.85%, 6.30%, 21.04%, and 1.81% through the XPS analysis. The high C content originates from the porous carbon spheres, whilst the N content of 6.3% is due to the condensation process, in which decomposition of the added ammonia formed N species. However, this low N content was sufficient to induce strong anchorage of the Ru NPs on the CSs. The actual Ru content was nearly 1.81%, indicating the successful confinement of Ru NPs in the CSs. The atomic % of O being 21.04% signifies the introduction and activation of oxygen vacancies that enhance the electron transfer and electrical conductivity of Ru–CS material.

### 3.2. Electrochemical Performance of the Sensors

**Figure 4:**
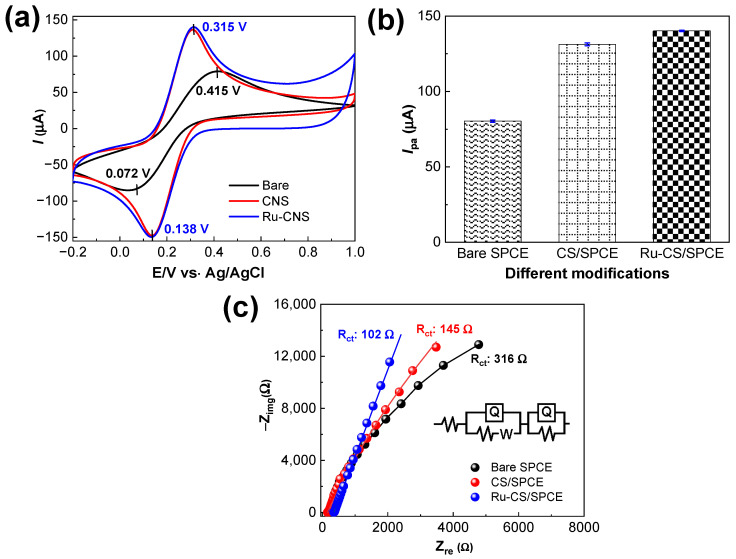
(**a**) CV data for bare the SPCE, CS/SPCE, and Ru–CS/SPCE in the 5 mM Fe(CN)_6_]^3−/4−^ redox couple prepared in 0.1 M KCl, at a 50 mV s^−1^ scan rate. (**b**) *I*_pa_ plot. (**c**) EIS profiles of the sensors in the redox couple. The inset is the fitted equivalent Randles circuit, *R*(*Q*(*RW*))(*QR*).

The electrochemical properties of the fabricated sensors were evaluated through a cyclic voltammetry (CV) analysis in 5.0 mM Fe(CN)_6_]^3−/4−^ in 0.1 M KCl electrolytes at a scan rate of 50 mV s^−1^. Figure 4a shows the CV data of bare the SPCE, CS/SPCE, and Ru–CS/SPCE. The CV curves of the three sensors exhibited well-resolved redox peaks, indicating efficient electron-transfer kinetics. The anodic peak potential (*E*_pa_) of the bare SPCE was determined as 0.415 V. Successive modifications of the SPCE with CSs and Ru-CSs negatively shifted the *E*_pa_ to 0.315 V. The remarkable negative shift of the *E*_pa_ indicates the efficient electrocatalytic activity of the Ru–CS/SPCE. The peak-to-peak separation (Δ*E*p) of the aforementioned sensors was 0.34, 0.18, and 0.17 V, respectively. The low Δ*E*p value indicates the fast electron-transfer properties due to the successive Ru–CS modifications. However, the anodic peak current (*I*_pa_) for the Ru–CS/SPCE was 140.2 μA, which is 1.7-fold higher than that of the bare SPCE (80.35 μA) and 1.1-fold higher than that of the CS/SPCE (131.26 μA), specifying that Ru–CS impregnation enhanced the conductive properties of the sensor (Figure 4b). Along with the well-resolved redox peaks in the spectrum of the Ru–CS/SPCE, the non-redox/pseudocapacitive currents were also substantially enhanced in the potential window of 0.3–1.0 V, which may be attributed to the high-valence Ru redox center [39].

The resistances of the sensors were calculated from the EIS data. Figure 4c shows the Nyquist diagrams from the EIS profiles within the 100 kHz to 0.1 Hz frequency range under the alternative current amplitude of 0.005 V. The overall resistance is determined by the resistance of the solution (*R_s_*) and the internal resistance produced by Ru–CSs. Figure 4c reveals that at the sensor surface, diffusion occurs at low frequencies, whereas resistance occurs at higher frequencies. The high charge–transfer resistance (*R_ct_*) of 316 Ω corresponds to the bare SPCE, implying high resistance in this sensor. Fitting the data with the Equivalent Randles circuit, *R*(*Q*(*RW*))(*QR*), demonstrated lower *R_ct_* values of 145 and 102 Ω for the CS/SPCE and Ru–CS/SPCE, respectively. The Ru–CS/SPCE exhibited the lowest resistance, which explains the diffusion-controlled electron transfer in this system.

Figure S2a shows the electrochemical outputs of the Ru–CS/SPCE at various scan rates in 5.0 mM Fe(CN)_6_]^3−/4−^. As the scan rate increased, sharp and well-defined redox peaks, with gradual increases in *I*_pa_ and *I*_pc_, were observed. The linear plots of *I*_pa_ and *I*_pc_ as a function of the square root of the scan rate indicated diffusion-controlled electron-transfer kinetics at the Ru–CS/SPCE surface (Figure S2b). Electron transfer at the sensors in the Fe(CN)_6_]^3−/4−^ redox couple is a reversible process. The electrochemically active surface area (ASA) was determined using the Randles–Ševčík equation [4]. The ASA values of the bare SPCE, CS/SPCE, and Ru–CS/SPCE were 0.1274, 0.1439, and 0.1590 cm^2^, respectively. The ASA of CS/SPCE and Ru–CS/SPCE was 1.20- and 1.25-times larger, respectively, than that of the bare SPCE. The larger ASA is due to the confinement of the Ru NPs in the CSs, which boosted the conductivity of the Ru–CS/SPCE.

The roughness factor (ASA vs. geometrical area ratio) was 1.26. This roughness factor is related to the presence of Ru–CSs, which increases the mass transport rate of the sensor. The heterogeneous rate constant (k^0^) was computed using the Nicholson equation, where the working curves relate Δ*E*_p_ to a kinetic parameter (Ψ). k^0^ was calculated as previously described [10]. The calculated k^0^ was 5.0 × 10^−3^ cm^2^ s^−1^, indicating rapid electron transfer at the sensor.

**Figure 5:**
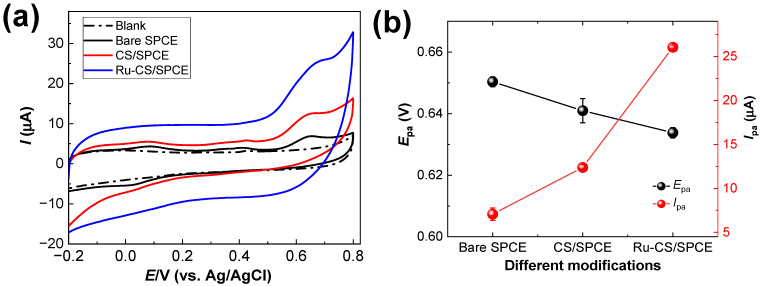
(**a**) CV data for modified sensors in 0.1 M phosphate buffer (pH 8.0) containing 10 µM MT. (**b**) Respective *E*_pa_ and *I*_pa_ plots.

The oxidation of MT at the bare SPCE, CS/SPCE, and Ru–CS/SPCE was ruled out using CV experiments. Figure 5a shows the CV graphs for the sensors in the presence of 10.0 μM MT in 0.1 M phosphate buffer (pH 8.0). A small broad irreversible oxidation peak for MT was observed at +0.65 and 0.64 V with bare the SPCE and CS/SPCE, respectively. The *I*_pa_ values generated at the bare SPCE and CS/SPCE were 7.06 and 12.38 μA, respectively. For Ru–CS/SPCE, the oxidation peak for MT was observed at 0.63 V, with a higher *I*_pa_ value of 26.05 μA (Figure 5b). The slight negative shift of the *E*_pa_ value and the 3.7-fold increase in the *I*_pa_ value of the Ru–CS/SPCE (26.05 μA) indicate that Ru–CSs are effective for the electrocatalysis of MT.

### 3.3. Effect of Scan Rate on the Ru–CS/SPCE

**Figure 6:**
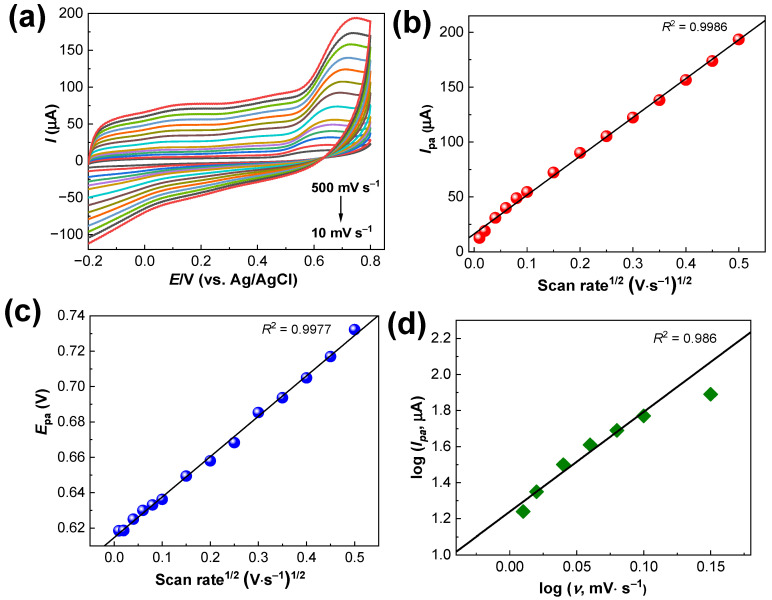
(**a**) CV data for the Ru–CS/SPCE at different scan rates from 10 to 500 mV s^−1^ (10, 20, 40, 60, 80, 100, 150, 200, 250, 300, 350, 400, 450 and 500 mV s^−1^) in 50 µM MT prepared in 0.1 M phosphate buffer (pH 8.0). (**b**) *I*_pa_ and (**c**) *E*_pa_ plots from data in (**a**). (**d**) log (*I*) vs. log (ν) plots of the *I*_pa_ up to a 100 mV scan rate.

Figure 6a shows the electrochemical data for the Ru–CS/SPCE at different scan rates in 50.0 μM of MT prepared in phosphate buffer solution (pH 8.0). When the scan rate was increased from 10 to 500 mV s^−1^, well-defined oxidation peaks were recorded, with increasing *I*_pa_ values. The high ion diffusion rate at higher scan rates resulted in an increased response. As shown in Figure 6b, the linear dependence of *I*_pa_ on the square root of the scan rate implies diffusion-controlled electron transfer at the Ru–CS/SPCE. Figure 6c shows the plots of *E*_pc_ vs. square root of the scan rate; the obtained linear regression equation is as follows:

*E*_pa_ = 0.2284x ± 0.315 × log*v*, *R*^2^ = 0.999

The electron-transfer rate constant (K_s_) was estimated using Laviron’s derivation given below [4]:

*E*_p_ = *E*_0_ + (2.303R*T*/*αn*F) log(R*T*K_0_/*αn*F) + (2.303R*T*/*αn*F)log*v* (1)

Here, *E*_p_ and *E*_0_ are the anodic and formal potentials; R is the universal gas constant, *T* is the temperature, *α* is the electron transfer coefficient, F is the Faraday constant, and *n* is the number of electrons involved. By substituting the obtained slope and intercept values into Equation (1), the values of *α* and K_s_ were calculated to be 0.9 and 2.1 s^−1^, respectively. Thus, two electrons are transferred during the reaction.

Typically, the diffusion-controlled or capacitive-controlled reaction mechanism at the sensor–electrolyte interface can be distinguished from the power law equations as below:

*I* = aν^b^ (2)

where *I* signifies the current, a is a constant, ν is the scan rate, and b is the calculated value close to 0.5 or 1. A b-value of 0.5 suggests diffusion-controlled processes, while a b-value of 1 illustrates the capacitive type of the reaction mechanism. Here, the calculated b-value for the *I*_pa_ was 0.63 (Figure 6d) suggesting a discrete/mixed reaction mechanism, with the predominance of a diffusion-controlled mechanism. The contribution of capacitive-controlled (k_1_ν^1/2^) and diffusion-controlled processes (k_2_ν) could be analyzed using the given Equation (3).

*I*(V) = k_1_ν^1/2^ + k_2_ν (3)

where *I*(V) is the current at potential V and ν is the scan rate. “k_1_” and “k_2_” are constants, and “k_1_ν” and “k_2_ν” are related to the capacitive and diffusion-controlled process, respectively [40]. Thus, at lower scan rates (10–100 mV s^−1^), the linear relationship of the log current (*I*) and log scan rate (ν) plots describes the dominance of diffusion-controlled mechanisms occurring at the sensor. However, at higher scan rates, the diffusion-controlled process likely decreases.

### 3.4. Optimization of Solution pH

**Figure 7:**
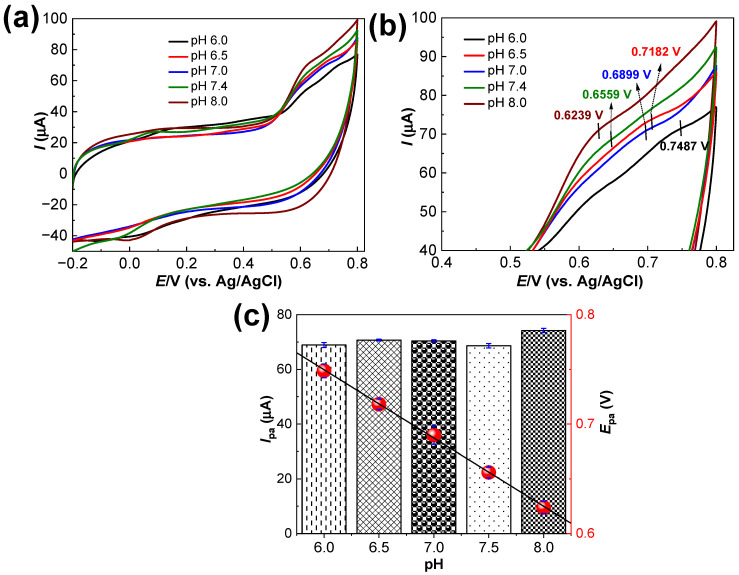
(**a**) CV responses of the Ru–CS/SPCE in 0.1 M phosphate buffer of different pH (6.0–8.0) containing 40 µM MT. (**b**) Magnified region of CV within the potential window of 0.4–0.8 V. (**c**) Corresponding *I*_pa_ and *E*_pa_ plots.

The optimum electrolyte pH is important for determining the *E*_pa_ and *I*_pa_ values for the electrocatalytic oxidation of MT at the Ru–CS/SPCE sensor. CV analysis in the presence of 40.0 μM MT was performed in the pH range of 6.0–8.0 at 50 mV s^−1^ (Figure 7a). The magnified region of the same voltammogram is presented in Figure 7b, showing that the *E*_pa_ shifted toward the less positive region as the solution pH increased (up to pH 8.0). This *E*_pa_ shift is due to the participation of a proton in the electrode reaction. Moreover, good linearity between the *E*_pa_ and pH was observed (Figure 7c). The *I*_pa_ responses plotted in Figure 7c illustrate a high *I*_pa_ value of 72.24 μA at pH 8.0. The CV responses evince a slope of −0.31 mV/pH. Thus, the participation of two electrons and one proton (2e^−^; H+) in the oxidation of MT to quinone imine is substantiated. 

**Figure 8:**
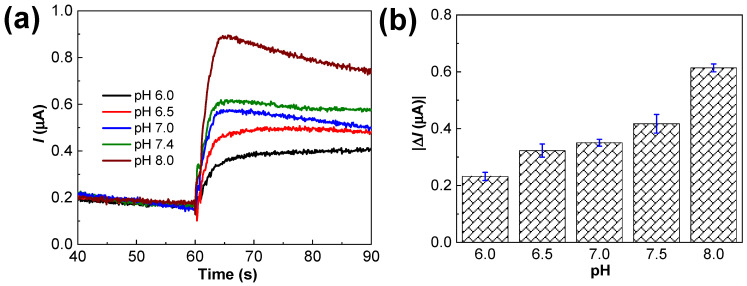
(**a**) Amperometric responses of the Ru–CS/SPCE in 0.1 M phosphate buffer of different pHs (pH 6.0–8.0) containing 5.0 µM MT, at the applied potential of +0.6 V. (**b**) Plot of current values determined from (**a**).

The optimum pH was verified using the CA technique, as displayed in Figure 8a. Similar to the CV data, at pH 8.0, higher current responses (0.614 μA) were obtained with 5.0 μM MT addition than at the other pH values (Figure 8b). Phosphate buffer solution (pH 8.0) was selected as the solution with the optimal pH.

### 3.5. Optimization of Working Potential; Selectivity and Sensing Mechanism at the Ru–CS/SPCE

Compounds with structures similar to that of the analyte often pose a risk of interference. Several biomolecules could potentially interfere with MT detection, and hence testing through CA experiments is essential. AA is electrochemically active and is oxidized at 0.1 V (vs. Ag/AgCl). NE, EP, and DA are highly electroactive catecholamines; however, these compounds are oxidized at a potential of 0.15 V. Since MT is oxidized at higher potentials (above 0.6 V), these compounds could substantially interfere with MT detection [41]. Also, 5-HT is structurally similar to MT and is a metabolic precursor of MT [42,43,44]. Thus, MT precursors or metabolites are also expected to interfere with the analysis of MT in pharmaceutical samples.

**Figure 9:**
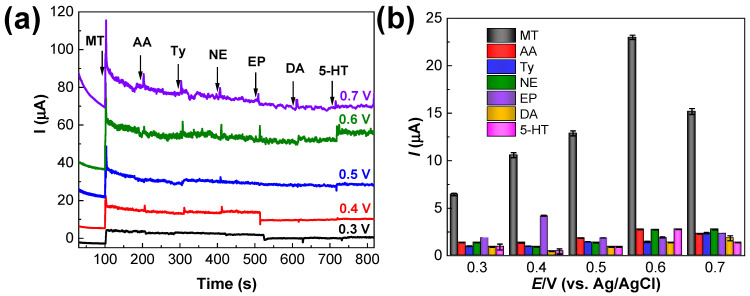
(**a**) Selectivity of the Ru–CS/SPCE in 0.1 M PBS at pH 8.0 with the addition of 40 µM MT and various interferents, such as AA, Ty (25-fold higher), NE, EP, DA, and 5–HT, at 400 µM (10-fold higher), under applied potentials from 0.3 to 0.7 V. (**b**) Plot of *I*_pa_ values 20 s after adding each analyte.

The selectivity of the Ru–CS/SPCE towards MT was determined in the presence of other interferents, such as AA, Ty, NE, EP, DA, and 5-HT. Interestingly, the sensor exhibited high selectivity towards MT, and only negligible interference was found when the concentrations of the tested interferents were ten-fold higher than that of MT (Figure 9a). It is considered that a substance caused interference at a concentration level that led to a change of 10% in the initial analytical signal of MT. According to this criterium, the maximum concentrations of each interferent that can produce interference were calculated from the CA responses (Figure 9a). The histograms in Figure 9b illustrate the high current response (22.99 µA) with the addition of MT at an applied potential of 0.6 V. The current response was lower at applied potentials of 0.3, 0.4, 0.5, and 0.7 V. The lowest responses were recorded at 0.3 V. In the potential range of 0.3–0.7 V, negligible interference (less than 8%) was recorded for the tested interferents, as illustrated in Figure 9b. The threshold concentrations of NE, EP, DA, and 5-HT were 400 µM. The concentrations higher than 400 µM could probably exert interferences in MT detection using the Ru–CS/SPCE sensor. AA and Ty even at 25-fold did not exert any interferences. Because the maximum current response for MT was recorded at an applied potential of 0.6 V (Figure 9b), this was chosen as a suitable working potential for the sensor in the presence of miscellaneous interfering compounds. Herein, the electrostatic interactions between the carbon ring of Ru–CSs and the benzene ring of MT plays an important role in improving the selectivity of the sensor towards MT.

**Scheme 2:**
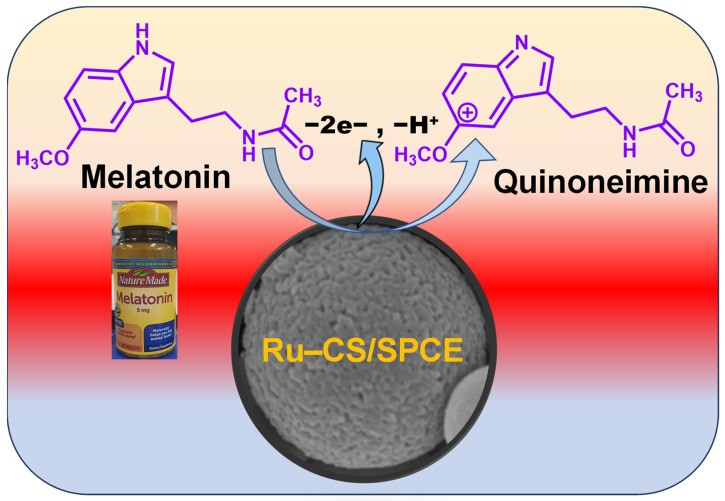
Mechanism of MT electro-oxidization to its corresponding quinoneimine taking place at the Ru–CS/SPCE sensor.

The proposed sensing mechanism of the Ru–CS/SPCE is as follows: first, MT molecules are adsorbed on the Ru–CS surface via π–π interactions through the conjugated carbon ring of Ru–CSs and the benzene ring of MT. The adsorbed MT is electro-oxidized to the corresponding intermediate quinoneimine ions (Scheme 2) [4]. At highly acidic and basic pHs, MT protonation is compromised, and at the near-neutral/optimized pH of 8.0, MT is easily oxidized to its quinoneimine form. Thus, the oxidation of MT relies solely on the electrostatic interactions between the carbon ring of Ru–CSs and the benzene ring of MT.

Briefly, CSs interact strongly with Ru ions to form ion–molecule adducts, which enhances the catalytic activity and chemical stability of Ru–CSs. The Ru NPs are anchored on the surfaces of CSs through non-covalent interactions, mainly π–π and hydrogen bonding interactions. The strong electronic interactions within Ru–CSs, mainly involving the Ru–O and Ru–C groups, contribute to accelerated electron transfer to and from MT.

Additionally, the introduction of electronegative polymers and molecularly imprinted polymers could actively repel the negatively charged interferents, thus eliminating the effects of interferences. However, completely eliminating the interferences of NE, EP, and DA is unavoidable and necessitates the introduction of advanced analytical techniques and/or specially tailored nanomaterials [45].

### 3.6. Amperometric Estimation of MT on the Ru–CS/SPCE

**Figure 10:**
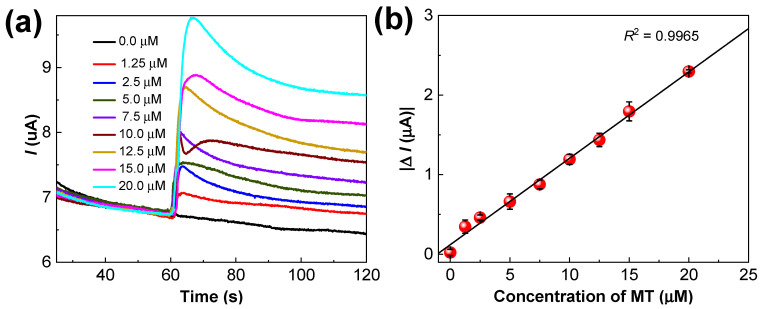
(**a**) Amperometric responses of the Ru–CS/SPCE in 0.1 M PBS pH 8.0 with different concentrations of MT under an applied potential of +0.6 V. (**b**) Corresponding linear calibration plot illustrating linearity from 1.25 µM to 20.0 µM MT.

CA measurements were performed using the Ru–CS/SPCE with various MT concentrations (0.0, 1.25, 2.5, 5.0, 7.5, 10.0, 12.5, 15.0, and 20.0 µM). The current response of the Ru–CS/SPCE increased rapidly with an increasing MT concentration (Figure 10a); the current responses measured after 20 s of MT addition are shown in the calibration plot in Figure 10b. The error bars in the calibration plot indicate the standard deviations of four mean values (Figure 10b). From the obtained calibration plot, the linear dynamic range was 1.25–20.0 µM, with a sensitivity of 0.85 µA µM^−1^ cm^−2^. The limit of detection (LOD) was 0.27 µM (S/N = 3), which is much lower than that of reported amperometric MT sensors [10,46] and the other electrochemical sensors summarized in Table 1.

### 3.7. Reproducibility and Stability

The reproducibility of the Ru–CS/SPCE sensor (*n* = 4) for MT (5.0 µM) detection was tested using CA experiments. The relative standard deviation obtained for the four identically prepared sensors was 4.1%. The stability of the sensors was examined over the course of four weeks, in which the current response of the sensor with MT addition was 98.8%, 89.5%, 85.9%, and 82.9% after each successive week, evidencing the good stability of the sensor (Figure S3).

### 3.8. Analysis of Pharmaceutical Samples

The analytical usefulness of the Ru–CS/SPCE was evaluated by using the regular addition method to determine the amount of MT in pharmaceutical samples. All analyses were performed in triplicate, and the mean values, standard deviations, and relative standard deviations were calculated. The CA data for the pharmaceutical samples (Table 2) demonstrated acceptable recovery percentages of ~100%, with a low relative standard deviation of less than 3.0% compared to the label value. Hence, the fabricated sensor is a simple and sensitive tool for determining the MT concentration in pharmaceutical samples.

## 4. Conclusions

Ru–CS materials that readily interact with MT via strong electronic interactions were synthesized. The formation of ion–molecule adducts between the CSs and Ru ions enables efficient adsorption of Ru in the CS matrix. Ru NPs confined in CSs showed good electron-transfer kinetics, resulting in an increased current response towards MT. The improved electrochemical performance of the Ru–CS/SPCE is attributed to its high ASA of 0.1590 cm^2^. The Ru–CS/SPCE exhibited a linear response in the MT concentration range of 1.25–20.0 µM, with a sensitivity of 0.85 µA µM^−1^ cm^−2^ and a much lower LOD of 0.27 µM (S/N = 3). The Ru–CS/SPCE exhibited high selectivity for MT in the presence of ten-fold higher concentrations of structurally similar interferents and other biomolecules. In the pharmaceutical samples, the Ru–CS/SPCE exhibited satisfactory recovery percentages with low relative standard deviations, indicating precise measurement by the sensor. The low LOD of the sensor and the lack of sample separation prior to analysis are important characteristics that enable the rapid detection of MT in complex samples. These results suggest that the fabricated Ru–CS/SPCE is a highly applicable tool for the quality control of medicines at a low cost and offers the potential for automation. Furthermore, the simple fabrication process makes it a potential electrochemical platform for developing new point-of-care systems for detecting MT.

## Figures and Tables

**Table 1:** Performance comparison of the Ru–CS/SPCE with previously reported sensors in MT detection.

Electrochemical Sensor	Linear Range (µM)	LOD (µM)	Electrochemical Technique	Tested Real Samples	Reference
CPE	3.0–550	2.3	CV, LSV	Pharmaceutical samples	[47]
BDD	0.5–4.0	0.11	CV, SWV	Pharmaceutical and human urine samples	[19]
BT-drug ion pair PVC sensor	1.0–1000	7.0	Potentiometry	Pharmaceutical samples	[48]
GCE	10.0–500	1.48	CV, DPV, SWV	Pharmaceutical and human serum samples	[49]
ZnFe_2_O_4_/CPE	50.0–500	3.0	SWV	Fruits, human urine, and pharmaceutical samples	[50]
Graphene/SPCE	1.00–300	0.87	CA	Pharmaceutical samples	[10]
Gr-Av	10.0–100.0	0.47	SWV	Synthetic urine, human blood serum, and pharmaceutical samples	[51]
GO nanoribbons/SPCE	5.0–3000	1.1	CV, DPV	Pharmaceutical samples and herb extracts	[52]
Carbon disc electrode	2.5–1000	1.3	CE-ED	Pharmaceutical samples	[53]
MnHCF-PEDOT/GCE	100–4600	100	CV, CA	–	[46]
Polymer-PdNPs-rGO/GCE	5.0–100	0.09	CV, SWV	Pharmaceutical and human urine samples	[54]
rGO/RuO_2_/GCE	2.0–20.0	0.18	DPV	–	[42]
Ru–CS/SPCE	1.25–20.0	0.27	CV, CA	Pharmaceutical samples	This study

CPE: carbon paste electrode; LSV: linear sweep voltammetry; BDD: boron-doped diamond electrode; SWV: square wave voltammetry; BT: bismus tetraiodate; PVC: poly vinyl chloride; GCE: glassy carbon electrode; DPV: differential pulse voltammetry; ZnFe_2_O_4_: zinc ferrite; Gr-Av: graphite automotive varnish; GO: graphene oxide; CE-ED: capillary electrophoresis with electrochemical detection; MnHCF: manganese hexacyanoferrate; PEDOT: mixed-valent poly(3,4-ethylenedioxythiophene); PdNPs: palladium nanoparticles; rGO: reduced graphene oxide; RuO_2_: ruthenium oxide.

**Table 2:** Comparison of MT contents (mean of three replicates) in pharmaceutical samples using the CA technique.

Pharmaceutical Samples	MT Taken (mg)	MT Found (mg)	Recovery (%)	RSD (%)
Nature Made MT	5.0	4.99	97.4	1.1
	10.0	9.63	95.2	2.9
	15.0	14.9	97.6	2.1
	20.0	19.03	94.6	0.7
Melatonina (Cosmopharm)	3.0	3.11	102.2	2.9
	6.0	6.13	101.4	2.3
	9.0	9.09	100.6	1.6
	12.0	12.10	102.0	2.6

RSD: relative standard deviation.

## Data Availability

Not applicable.

## Supplementary Materials

The following supporting information can be downloaded at: https://www.mdpi.com/article/10.3390/bios13100936/s1, Section S1: Instrumentation and Measurements. Figure S1: (a) The XPS survey spectra of Ru–CS/SPCE and (b) the deconvoluted Ru3p spectra evincing two distinct sub-peaks Ru3p_1/2_ and Ru3p_3/2_ with mixed valence states Ru^0^ and Ru^2+^ of metallic Ru. Figure S2. (a) CV data for Ru–CS/SPCE in 5 mM [Fe(CN)_6_]^3−/4−^ prepared in 0.1 M KCl at increasing scan rates (10 to 200 mVs^−1^) and (b) respective *I*_pa_ and *I*_pc_ calibration plots. Figure S3. (a) Amperometric current responses of Ru–CS/SPCE measured and plotted against 20.0 µM concentration of MT under an applied potential of +0.6 V. (b) Relative % of current responses retained in the 0–4 weeks period.
