# Supplementary material for: Ruthenium-Anchored Carbon Sphere-Customized Sensor for the Selective Amperometric Detection of Melatonin
Source: Biosensors (Basel). 2023 Oct 18;13(10):936. doi: 10.3390/bios13100936 (PMC10605478; doi:10.3390/bios13100936)
Supplement: Supplementary file 1 [file biosensors-13-00936-s001.zip › biosensors-2616068-supplementary.pdf]

Supplementary material

Ruthenium-Anchored Carbon Sphere-Customized Sensor for the Selective Amperometric Detection of Melatonin

Sivaguru Jayaraman[1], Thenmozhi Rajarathinam[1], Hyeon-Geun Jang [1], Dinakaran Thirumalai[2], Jaewon Lee[2,3], Hyun-jong Paik[4] and Seung-Cheol Chang [1]*

[1]Department of Cogno-Mechatronics Engineering, College of Nanoscience and Nanotechnology, Pusan National University, Busan 46241, Republic of Korea

[2]BIT Convergence-based Innovative Drug Development Targeting Metainflammation, Pusan National University, Busan, 46241, Republic of Korea

[3]Department of Pharmacy, College of Pharmacy, Pusan National University, Busan, 46241, Republic of Korea

[4]Department of Polymer Science and Engineering, Pusan National University, Busan 46241, Republic of Korea

*Correspondence: s.c.chang@pusan.ac.kr

S.1. Instrumentation and Measurements

Morphological analyses of the synthesized materials were performed by field-emission scanning electron microscopy (FE–SEM; Zeiss GEMINI500, (Carl Zeiss Microscopy Deutschland GmbH., Oberkochen, Germany) coupled with energy dispersive X–ray spectroscopy (EDX). High-resolution transmission electron microscopy (HRTEM) images were acquired using a HITACHI H–7600 200 kV instrument (Hitachi High–Tech Corp., Tokyo, Japan). The surface chemical composition of the was characterized by an X-ray photoelectron spectrometer (Thermo Scientific Company, USA). The average particle size was determined by dynamic light scattering (DLS), using particle size analyzer (Beckman Coulter, Brea, CA, USA). Cyclic voltammetry (CV) and electrochemical impedance spectroscopy (EIS) analyses were performed using a potentiostat (604E, CH Instruments, Inc., Austin, TX, USA). Chronoamperometry (CA) was performed using an electrochemical instrument (Compactstat.h Standard, Ivium Technologies, Eindhoven, Netherlands) interfaced with a personal computer.

A 2 mL disposable electrochemical cell was constructed for all electrochemical experiments. The sensor was placed in 2 mL of 0.1 M KCl solution containing 5.0 mM $[Fe(CN)_6]^{3-/4-}$, and the potential was swept from −0.2 to +1.0 V. EIS measurements were performed in the frequency range of 100 kHz and 0.1 Hz at open-circuit potential. For CA, a 2 mL disposable well with phosphate buffer solution was set up, and the sensor was polarized under a fixed potential of 0.60 V. Finally, the calibration curves for MT were generated; the standard deviation was determined from more than four values.

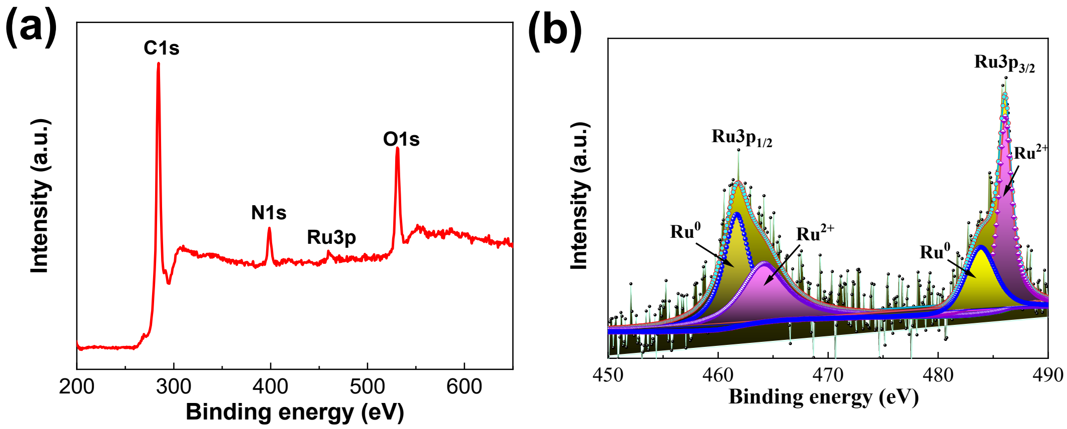

Figure S1. (a) The XPS survey spectra of Ru–CS/SPCE and (b) the deconvoluted Ru3p spectra evincing two distinct sub-peaks $Ru3p_{1/2}$ and $Ru3p_{3/2}$ with mixed valence states Ru^0 and Ru^{2+} of metallic Ru.

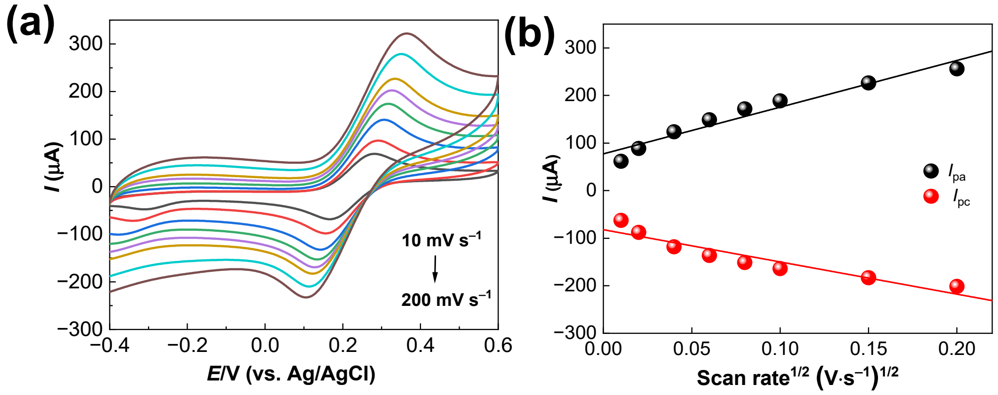

Figure S2. (a) CV data for Ru–CS/SPCE in 5 mM $[Fe(CN)_6]^{3-/4-}$ dissolved in 0.1 M KCl at increasing scan rates (10 to 200 mVs^{-1}) and (b) respective I_{pa} and I_{pc} calibration plots.

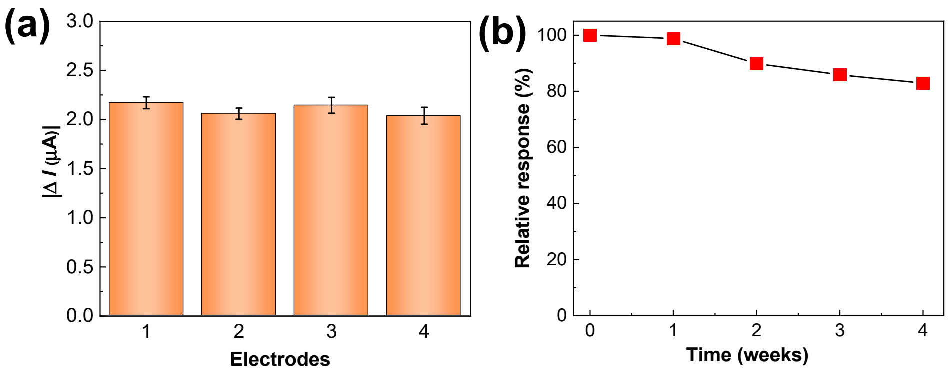

Figure S3. (a) Amperometric current responses of Ru–CS/SPCE measured and plotted against 20.0 μM concentration of MT under an applied potential of +0.6 V. (b) Relative % of current responses retained in the 0–4 weeks period.
